# The association and significance of H3K27me3 and a folate metabolic gene ACat2 in neural tube defects

**DOI:** 10.1186/s12937-016-0212-7

**Published:** 2016-11-03

**Authors:** Sifan Zhai, Mingzuo Zhao, Changcheng Zhou, Fenggang Lu, Huankai Zhang, Li Na, Shanshan Feng, Xiaoxin Qiang, Yong Du

**Affiliations:** 1 Graduate School, Ningxia Medical University, Ningxia, China; 2 Department of Scientific Research, General Hospital of Ningxia Medical University, 804 South Victory Street, Yinchuan, Ningxia China; 3 Department of General Surgery, Suqian People’s Hospital, Suqian, Jiangsu China; 4 Tai’an Hospital of Traditional Chinese Medicine, Tai’an, Shandong China; 5 No. 215 Hospital, Sino Shanxi Nuclear Industry Group, Xi’an, Shangxi China; 6 Department of General Surgery, General Hospital of Ningxia Medical University, Ningxia, China

**Keywords:** Neural tube defects, Folic acid, Neural stem cells, Epigenetics, H3K27me3

## Abstract

**Aim:**

To study the association between the expression of H3K27me3 and ACat2 (a folate metabolic protein), in order to elucidate the protective mechanism of folic acid (FA) in neural tube defects (NTDs).

**Methods:**

Eighteen female SD rats were randomly divided into normal, NTD and FA group. NTD group was induced by all-trans retinoic acid (ATRA) at E10d. FA group was fed with FA supplementation since 2 weeks before pregnancy, followed by ATRA induction. At E15d, FA level in the embryonic neural tube was determined by ELISA. Neural stem cells (NSCs) were isolated. Cell proliferation was compared by CCK-8 assay. The differentiation potency was assessed by immunocytochemical staining. H3K27me3 expression was measured by immunofluorescence method and Western blot. ACat2 mRNA expression was detected by qRT-PCR.

**Results:**

Cultured NSCs formed numerous Nestin-positive neurospheres. After 5 days, they differentiated into NSE-positive neurons and GFAP-positive astrocytes. When compared with controls, the FA level in NTD group was significantly lower, the ability of cell proliferation and differentiation was significantly reduced, H3K27me3 expression was increased, and ACat2 mRNA expression was decreased (*P* <0.05). The intervention of FA notably reversed these changes (*P* <0.05). H3K27me3 expression was negatively correlated with the FA level (rs = −0.908, *P* <0.01) and ACat2 level (rs = −0.879, *P* <0.01) in the neural tube.

**Conclusion:**

The increased H3K27me3 expression might cause a disorder of folate metabolic pathway by silencing ACat2 expression, leading to reduced proliferation and differentiation of NSCs, and ultimately the occurrence of NTD. FA supplementation may reverse this process.

## Background

Neural tube defects (NTDs) are serious birth defects due to an abnormal opening in the spinal cord or brain from early embryo development which may cause mental retardation and permanent disability. It is the second most common birth defects with an incidence rate of 1.23‰, affecting approximately 300,000 births each year worldwide [1]. The disease is also associated with a high mortality and has become a serious healthy issue [2].

Folate is required for the production of new cells, especially for the synthesis of nucleic acids and proteins. It is also essential for the synthesis of S- adenosylmethionine (SAM), a primary methyl group donor for the methylation of proteins, DNAs, RNAs and lipids. Therefore, the folate biopathway plays an important role in cell proliferation, differentiation, transcriptional regulation of genes, etc. Recently, folate has become a research hotspot in NTDs [3–5]. It has been found that folate metabolic disorders affect the normal closure of neural tube [3, 6, 7], and the accumulation of Hcy, an intermediate metabolic product of folate, may cause the occurrence of NTDs [8, 9]. Numerous studies and clinical trials have shown that prenatal folic acid (FA) supplementation can reduce the risk of NTDs by approximately 70% [10–13]. Nevertheless, the protective mechanism of FA on NTDs remains unclear and needs to be clarified to provide a basis for its application in the prevention of the disease.

**Fig. 1:**
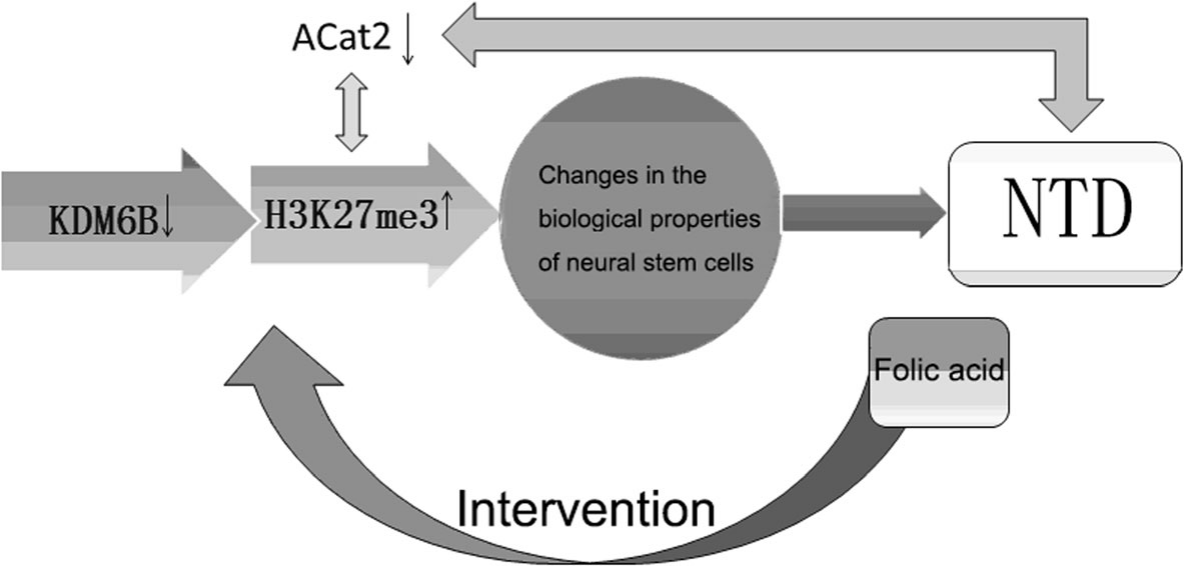
A pictorial representation of NTD pathway and associated genes such as ACat2 and H3K27me3. KDM6B, the H3K27me3 demethylase

Histone modification has been known as an important component of embryo epigenetics. As one of the most essential histone modification in the neuronal differentiation, H3K27me3 plays a vital role in the regulation of neuronal development [14]. A pictorial representation of NTD pathway and associated genes including histone H3 trimethyl Lys27 (H3K27me3) is shown in Fig. 1. During the closure of neural tube, the H3K27me3 demethylase JMJD3/KDM6B expression was increased, followed by the demethylation of H3K27me3, which promotes the differentiation of neural stem cells [15]. Acyl-coenzyme a-cholesterol acyltransferase 2 (ACat2) is a common membrane-bound enzyme that produces cholesteryl esters in eukaryotic cells [16]. Studies have detected a reduced ACat2 expression in hyperhomocysteinemia (HHcy) mice [17], suggesting that ACat2 might be related to the folate metabolic pathway through regulation on intracellular Hcy level. In our preliminary study, we have detected an association between ACat2 gene and H3K27me3 in NTD rats by ChIP-seq technology (unpublished data). We therefore speculate that an interaction might exist between H3K27me3 methylation and ACat2 expression during the development of NTDs.

Rats with all-trans retinoic acid (ATRA)-induced congenital neural tube defects has been widely used as a reliable and convenient NTD model [18, 19]. In this study, we evaluated the effect of FA on proliferation of neural stem cells (NSCs) in ATRA-induced NTD rats and analyzed the correlation between H3k27me3 expression and FA and ACat2 level, in order to investigate the mechanism of H3k27me3 in the occurrence of NTDs.

## Material and methods

### Animals

Mature SD rats weighting 260 ± 5 g were purchased from the Experimental Animal Center at the Ningxia Medical University (Certificate No.: SCXK (Ning 2011–0001)). Rats were mated (1 male vs.1 female) at midnight. Female rats were examined for yellowish vaginal discharge the next morning (Embryonic day 0, E0 d), and those with successful mating were devided into 3 groups: healthy control, NTD, and FA group. While FA group was fed daily with FA supplemtnation (60 μg / kg) since 2 weeks before preganancy, the other 2 groups were fed with regular food. NTD and FA groups were given a gavage dose of 90 mg / kg ATRA on E10d, and healthy control was given equal volume of olive oil. On E15d, adult rats in all groups were anesthesized by an intraperitoneal injection of chloral hydrate (3 ml/kg). Fetus was removed from the uterus under sterile conditions, and neural tube was obtained to isolate NSCs. Part of the neural tube was also stored at −80 °C. This study was approved by the Ethics Committee at the General Hospital of Ningxia Medical University.

### Determination of FA content

The frozen neural tube tissue was homogenized and centrifuged at 2500 rpm for 20 min. The supernatant was collected, and analyzed with rat FA ELISA kit (Chenglin Biotech., Beijing, China) following the manufature’s instructions. The optical density of each well was detected at the wavelength of 450 nm and the FA content was calculated based on the standard curve.

### Isolation and culture of spinal cord-derived NSCs

Fresh neural tube was cut into small pieces, trypsinized and prepared into 10^7^/ml cell suspension in serum-free complete medium (DMEM / F12 base medium containing 20 ng/ml EGF, 20 ng/ml bFGF, 2 % B27, 100 u/ml penicillin and streptomycin, Gibco, St. Louis, MO, USA). Cells were cultured at 37 °C in an incubator with 5 % CO2. Half of the medium was changed every other day with fresh medium. On day 5, the average number and diameter of neurospheres in 5 randomly selected visual field was counted under an inverted microscope (100 x) was recorded.

### Growth curve of NSCs

The single cell suspension of primary NSCs in each group was inoculated into 96-well plates and cultured at 37 °C, 5 % CO2 for 24 h. a total of 21 wells were prepared for each group. Three wells were measured using CCK-8 cell proliferation kit on each day from day 1 to 7. Specifically, cells were incubated with 10 μl of CCK solution (Beyotime, Shanghai, China) for 2 h and OD_450_ of each well was detected. The growth curve of each group was drawn.

### Immunocytochemical identification of primary and differentiated NSCs

The neurospheres were collected and cultured in a 24-well plate on a polylysine-coated (100 μl/ml) coverslip for 24 h. The medium was removed. Cells were fixed with 4 % paraformaldehyde for 20 min, treated in 1 g/l Triton × 100 for 20 min, and washed 3 times with PBS. The coverslip was blocked with goat serum at 37 °C for 20 min, and incubated with mouse anti-rat Nestin monoclonal antibody (1: 200 dilution) overnight at 4 °C. The coverslip was then washed with PBS and incubated with HRP-labeled goat anti-mouse polyclonal secondary antibody at 37 °C for 2 h. The coverslip was subjected to DAB color development for 20 s, and counterstained with hematoxylin for 1 min. The coverslip was washed with water, dehydrated with serial ethanol, treated with xylene, mounted in neutral gum, and observed under a microscope.

The neurospheres were also inoculated into a 24-well plate with a polylysine-coated coverslip, and cultured in NSC complete medium containing 10 % fetal bovine serum (Gibco) to induce differentiation. After 5 days, immunocytochemical identification was performed as described above using Nestin (1:200 dilution, Abcam, Cambridge, MA, USA), NSE (1:100 dilution, Boster, Wuhan, China), and GFAP (1:100 dilution, Boster) monoclonal antibody, respectively.

### Immunofluorescence and confocal microscopy

Cells at the exponential phase were cultured in a 24-well plate with a polylysine-coated coverslip for 24 h. Cells were incubated with mouse anti-rat H3K27me3 monoclonal antibody (1:200 dilution, Abcam) overnight at 4 °C, rinsed and incubated with goat anti-mouse fluorescent FITC-labeled secondary antibody (1:3000 dilution, Abcam) at 37 °C for 40 min. The coverslip was subjected to DAPI nuclear counterstain, treated with an anti-quenching agent, and mounted in neutral gum. The coverslip was then observed under a confocal laser fluorescence microscope. The mean number of cells with green fluorescence (target protein) in 5 randomly selected visual fields was recorded. The proportion of DAPI-positive cells indicated the relative expression of H3K27me3.

### Western blot analysis

Total protein of NSCs was extracted using a protein extraction kit and quantified using a BCA kit (Beyotime Institute of Biotechnology, Shanghai, China) according to the manufacture’s instruction. Equal amounts of total protein (20 μg) were separated by SDS-PAGE electrophoresis and transferred to polyvinylidene difluoride membranes. The membrane was blocked in TBS buffer with 5 % skim milk and 0.1 % Tween20 for 1 h, and incubated respectively with mouse anti-rat H3K27me3 monoclonal antibody (1:200 dilution, Abcam), mouse anti-rat GAPDH monoclonal antibody (1:1000 dilution, Abcam), mouse anti-rat ACAT2 monoclonal antibody (1:1000, Abcam), and mouse anti-rat β-tubulin monoclonal antibody (1:10000, Abcam) overnight at 4 °C. The membrane was then incubated with goat anti-mouse HRP-labeled secondary antibodies (1:2000 dilution, Abcam) at 37 °C for 2 h, and subjected to ECL detection for 5 min. The intensity of bands was detected by a Molecular Imager® ChemiDocTM XRS System (Bio-Rad Laboratories). The gray value of bands was analyzed by Image Lab 2.0 software (Bio-Rad Laboratories).

### Quantitative reverse transcription PCR (qRT-PCR) analysis

Total RNA of NSCs was extracted an E.Z.N.ATM MicroElute Total kit (Omega) and quantified using a spectrometer under a wavelength of 260 nm. Total RNA (500 ng/μl) was reverse transcribed into cDNA. PCR was performed using cDNA as template and the following primers: ACat2-forward: 5′-CCCGTGGTCATCGTCTCAG-3′, ACat2-reverse: 5′-GGACAGGGCACCATTGAAGG-3′; GAPDH-forward: 5′-AGCCACATCGCTCAGACA-3′; and GAPDH-reverse: 5′- TCTCCTGGGAGGCATAGACC-3′. The reaction condition was as follows: 95 °C 10 min, followed by 40 cycles of 95 °C 15 s, 61.9 °C 30 s and 72 °C 30 s. PCR products were analyzed by argarose gel electrophosis (2 %). The relative expression of ACat2 mRNA was calculated as the ratio of the grey value of ACat2 to that of GAPDH.

### Statistical analysis

All data were expressed as mean ± standard deviation and analyzed using IBM SPSS 20.0. Normally distributed data with homogeneity of variance was analyzed by one-way ANOVA. Data with non-normality or heterogeneity of variance was compared by Kruskal-Wallis H tests. Association between H3K27me3 and FA and ACat2 level was analyzed by Spearman rank correlation test. P values smaller than 0.05 are considered statistically significant.

## Results

### ATRA-induced NTD rats exhibited lower folic acid levels

**Fig. 2:**
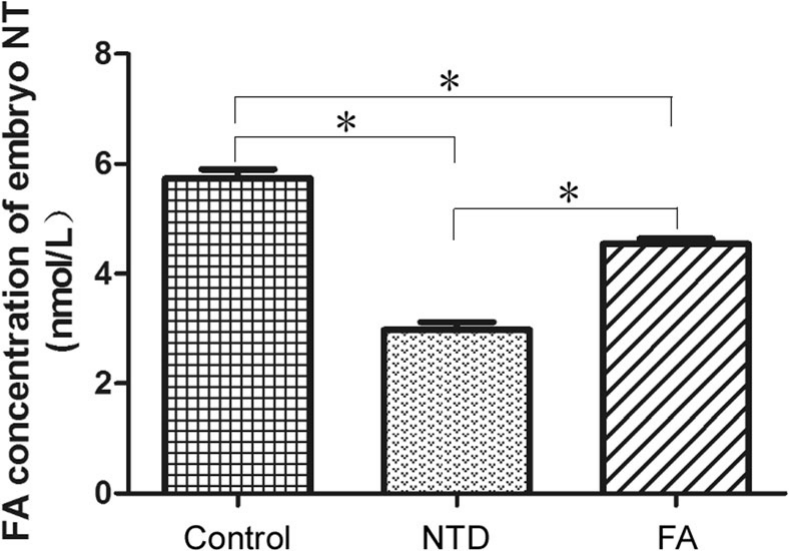
ELISA analyses comparing the FA concentration in embryonic neural tube tissue in three groups. ^*^, *P* < 0.05

The FA level in embryonic neural tube was compared by ELISA. As shown in Fig. 2, the FA level in NTD group was significantly reduced compared with healthy controls (*P* < 0.05). The FA level in FA group was significantly higher than that in NTD group (*P* < 0.05), although it was significantly lower compared with controls (*P* < 0.05).

### FA improved the impaired proliferation ability of NSCs in NTD

**Fig. 3:**
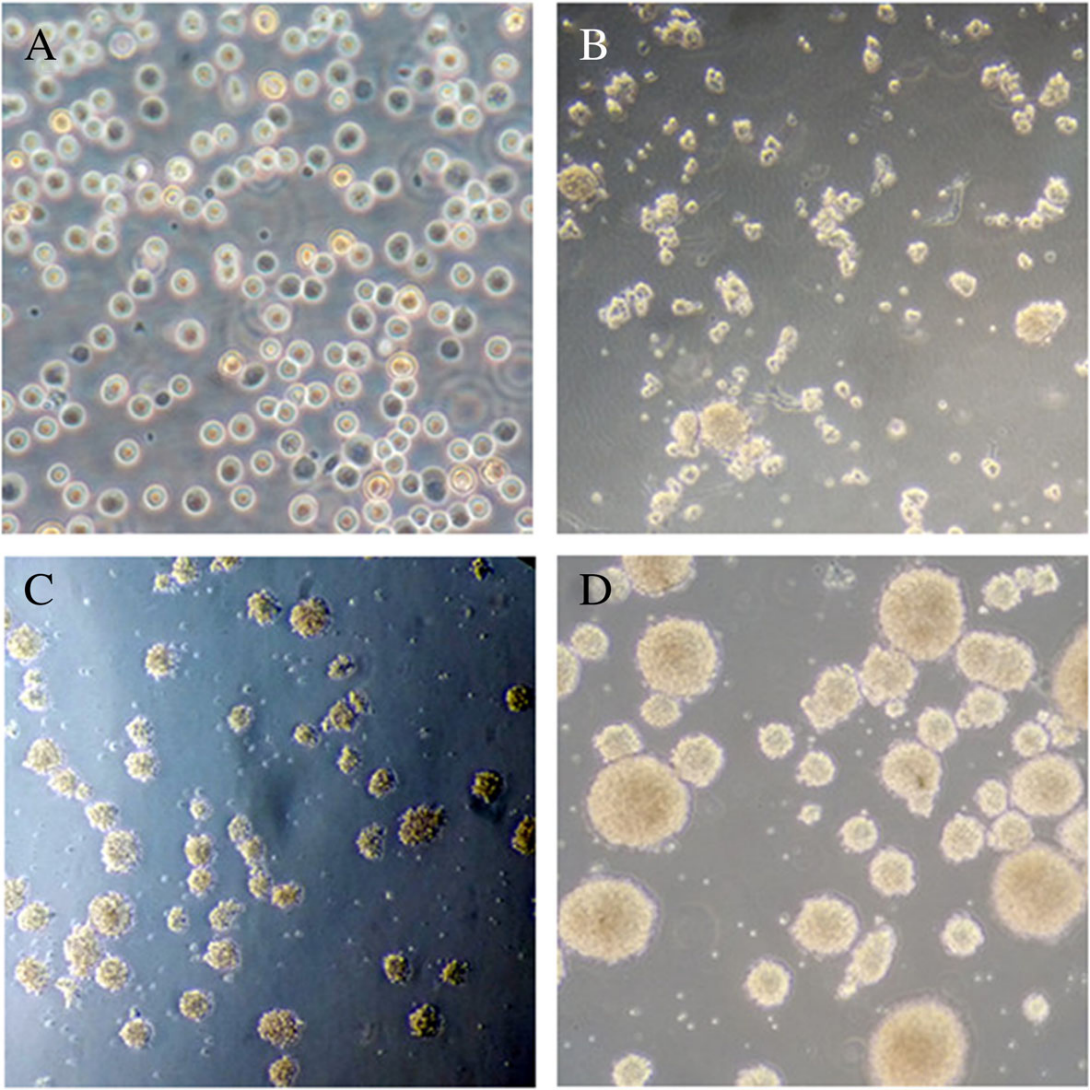
NSCs derived from neural tube were cultured in serum-free complete medium. **a**. In the beginning of culture, cells were small, round and translucent (100x). **b**: After 12 h, cells grew into small, round cell mass (200×). **c**: After 24 h, cell mass grew bigger and cell debris was observed (100×). **d**: On day 5, round cell mass grew into big neurosphere with cells well organized (200×)

**Fig. 4:**
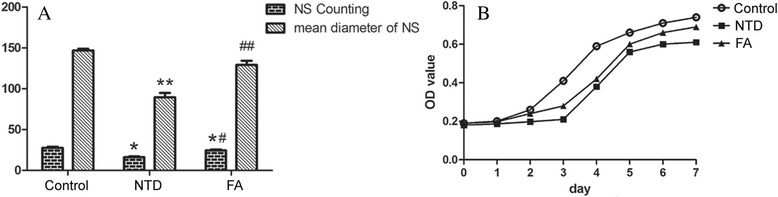
**a** Comparison of the average number and diameter of neurospheres in each group after 5-day culture in serum-free complete medium, suggesting a reduced proliferation of NTD-derived NSCs. Folic acid fortification significantly improved the cell proliferation. ^*^ *P* < 0.05, ^**^ *P* < 0.05, compared with normal controls. ^#^ *P* < 0.05, and ^##^ *P* < 0.01 compared with NTD group. **b** Growth curves of NSCs in three groups. Comparison of the average OD value in each group from day 1 to 7 shows lower cell proliferation in NTD-derived NSCs when compared with normal control (*P* < 0.05). Folic acid fortification significantly improved cell proliferation (*P* < 0.05)

NSCs in all groups were cultured in vitro to compare the proliferation ability. In the beginning of culture, cells were small, round and translucent (Fig. 3a). After 12 h, cells grew into small, round cell mass (Fig. 3b). After 24 h, cell mass grew bigger and cell debris was observed (Fig. 3c). On day 3, mulberry-shaped cell clusters was observed. On day 5, round cell mass grew into big neurosphere with cells well organized (Fig. 3d). The number of neurospheres in FA group (21.59 ± 3.48) was significantly higher compared with NTD group (17.91 ± 4.06, *P* < 0.05), but was significantly lower compared with healthy controls (26.93 ± 4.13, *P* < 0.05). Moreover, the mean diameter of neurospheres in FA group (137.74 ± 11.62) was also significantly larger compared with NTD group (90.31 ± 6.06, *P* < 0.01), despite that it was slightly smaller than that in controls (143.69 ± 12.20, Fig. 4a).

The cell proliferation of primary NSCs was further compared by CCK-8 assay. As shown in Fig. 4b, the NTD group exhibited lower activity of cell proliferation, whereas FA intervention notably improved the cell proliferation.

### FA improved the impaired differentiation ability of NSCs in NTD

**Fig. 5:**
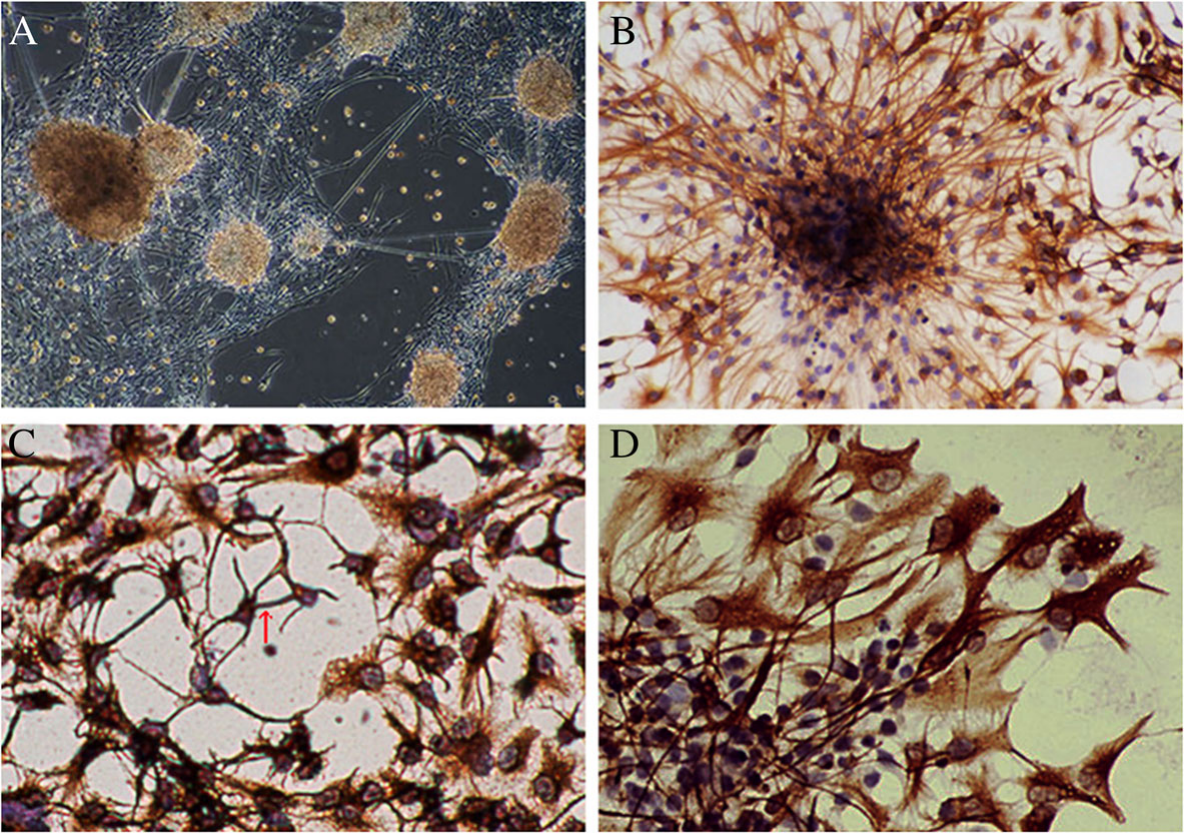
**a** Neurosphere exhibited adherent growth after several hours of incubation. Extensive thin, elongated projections were observed, connecting adjacent neurospheres into a complex network (100x); **b** Immunocytochemical staining showing primary NSCs with Nestin-positive cytoplasm and Nestin-negative nuclei (200x); **c** Small, round/oval/triangle NSE-positive neurons with thin and long projections were observed at day 5 since the differentiation induction (200x). Synaptic connections have been established between neurons (*red arrow*); and **d** large, irregular-shaped GFAP-positive astrocytes with multiple short and flat projections were observed at day 5 since the differentiation induction (200x)

**Fig. 6:**
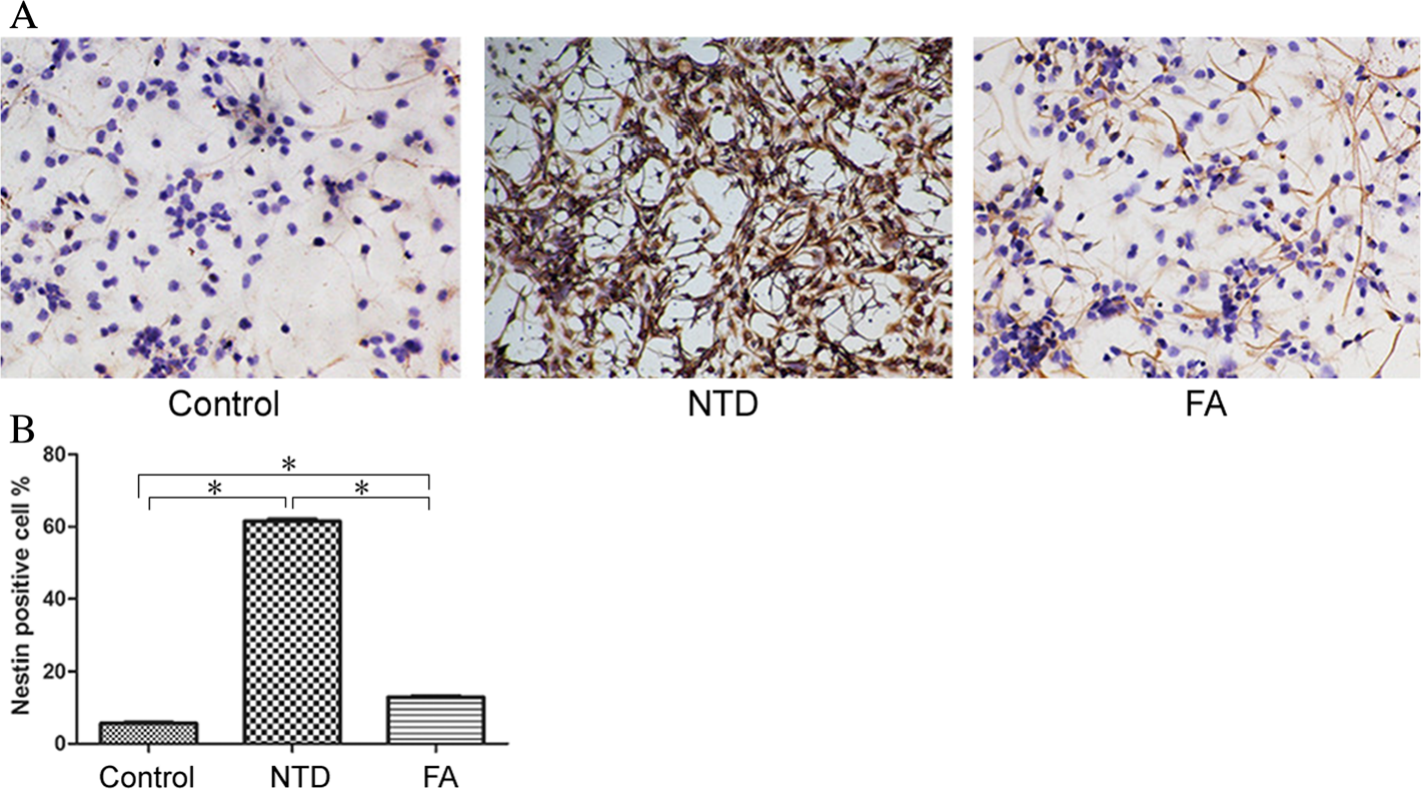
Immunocytochemical analyses of the remaining level of Nestin in cells after 5 days of differentiation induction. **a** Representative images of Nestin-postive cells in different groups; **b** Comparison of Nestin-positive cells in different groups by immunocytochemical staining. ^*^, *P* < 0.05

After several hours of culture, neurosphere exhibited adherent growth. Extensive thin, elongated projections were observed, connecting adjacent neurospheres into a complex network (Fig. 5a). Immunocytochemical analysis demonstrated the Nestin-positive cytoplasm and Nestin-negative nuclei, confirming the identification of NSC (Fig. 5b). After differentiation induction by FBS, small, round/oval/triangle NSE-positive neurons with thin and long projections (Fig. 5c) and big, irregular-shaped GFAP-positive astrocytes with multiple short and flat projections (Fig. 5d) were observed, indicating the multipotent differentiation potential of NSCs. In addition, as shown in Fig. 6, the remaining Nestin level in FA group (12.92 ± 0.48 %) was significantly lower compared with NTD group (61.52 ± 1.10 %, *P* <0.05), but was much higher than that in control group (5.64 ± 0.59 %, *P* <0.05), suggesting that FA supplementation markedly improved the impaired differentiation ability of NSCs in NTD.

### H3K27me3 expression was negatively correlated with FA level in the embryonic neural tube

**Fig. 7:**
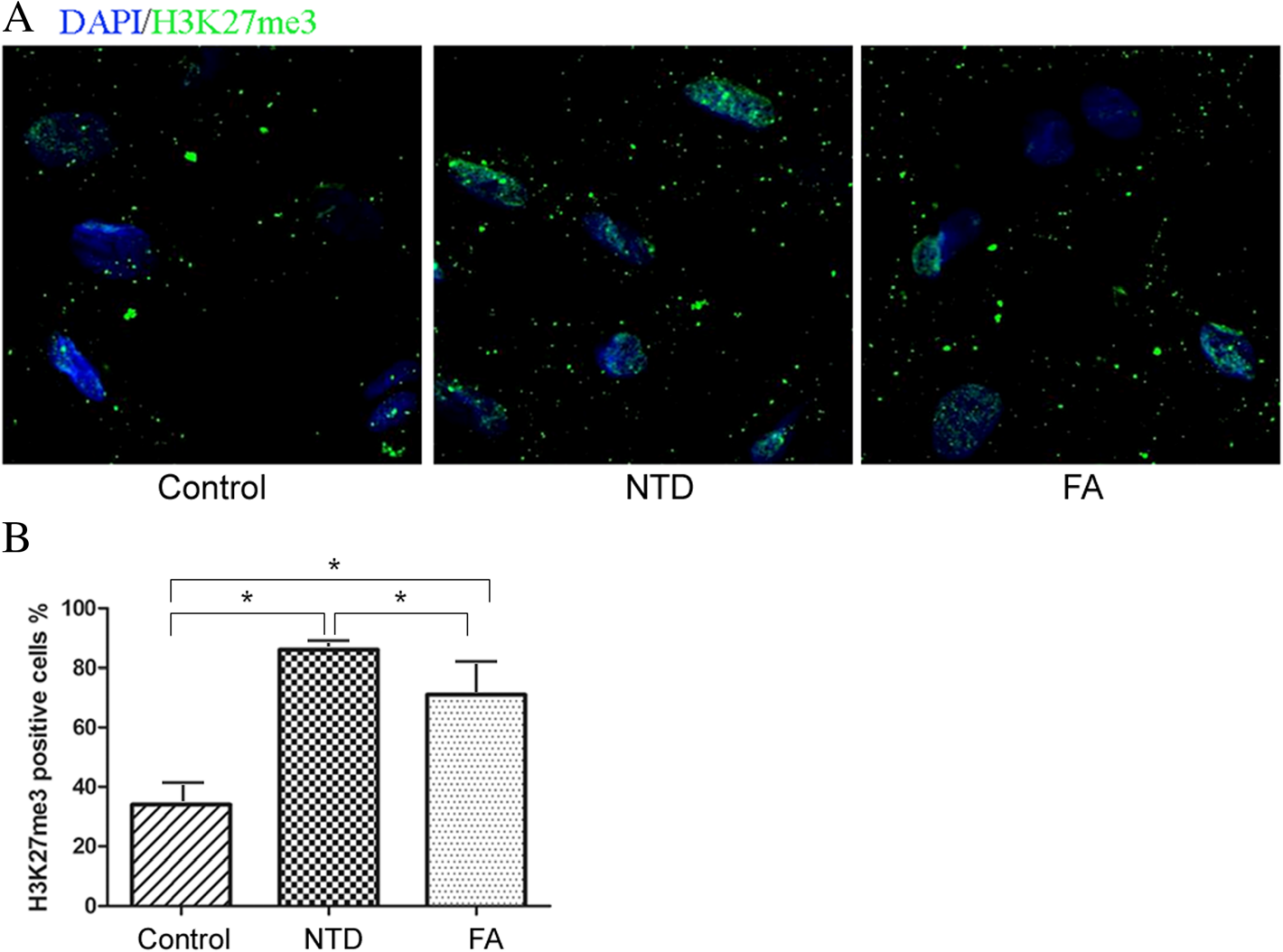
Detection of H3K27me3 expression by immunofluorescence and confocal microscopy. **a** representative images of NSCs under a confocal microscopy (200x). Target protein was observed as green fluorescence, and nuclei were stained as blue-fluorescent DAPI. **b** Comparison of H3K27me3-positive cells in different groups. ^*^, *P* < 0.05

**Fig. 8:**
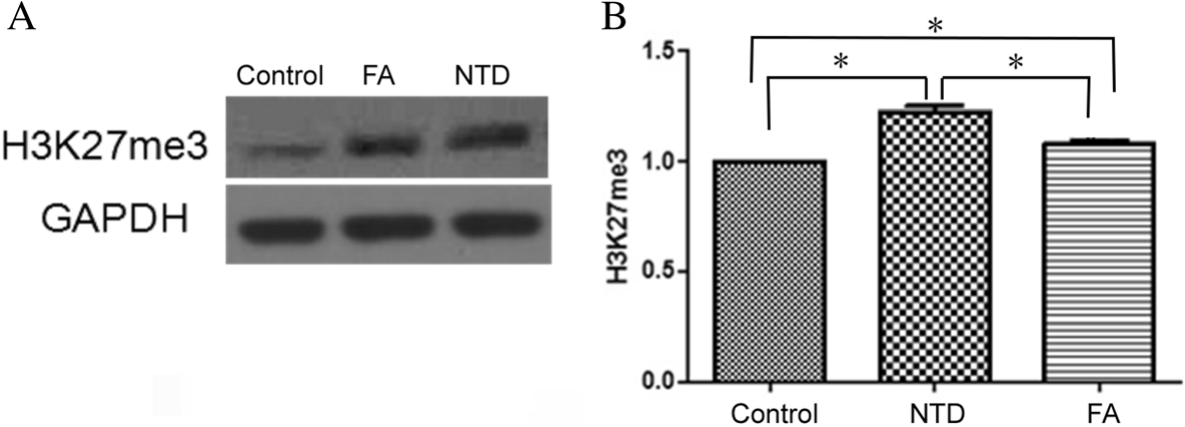
Western blot analyses comparing the H3K27me3 expression in NSCs in 3 groups. **a** Representative image of Western blot analysis ofH3K27me3 expression; **b** Quantification of the relative expression levels of H3K27me3

The expression of H3K27me3 was detected by immunofluorescence and confocal microscopy (Fig. 7a). Although H3K27me3 was expressed in all 3 groups, the H3K27me3 level in NTD group was the highest, followed successively by FA and control group (*P* <0.05, Fig. 7b). The result was consistent with further Western blot analysis (Fig. 8). Spearman rank correlation test revealed a negative correlation between H3K27me3 expression and FA level in the embryonic neural tube (rs = −0.908, *P* <0.01).

### H3K27me3 expression was negatively correlated with ACat2 level in the embryonic neural tube

**Fig. 9:**
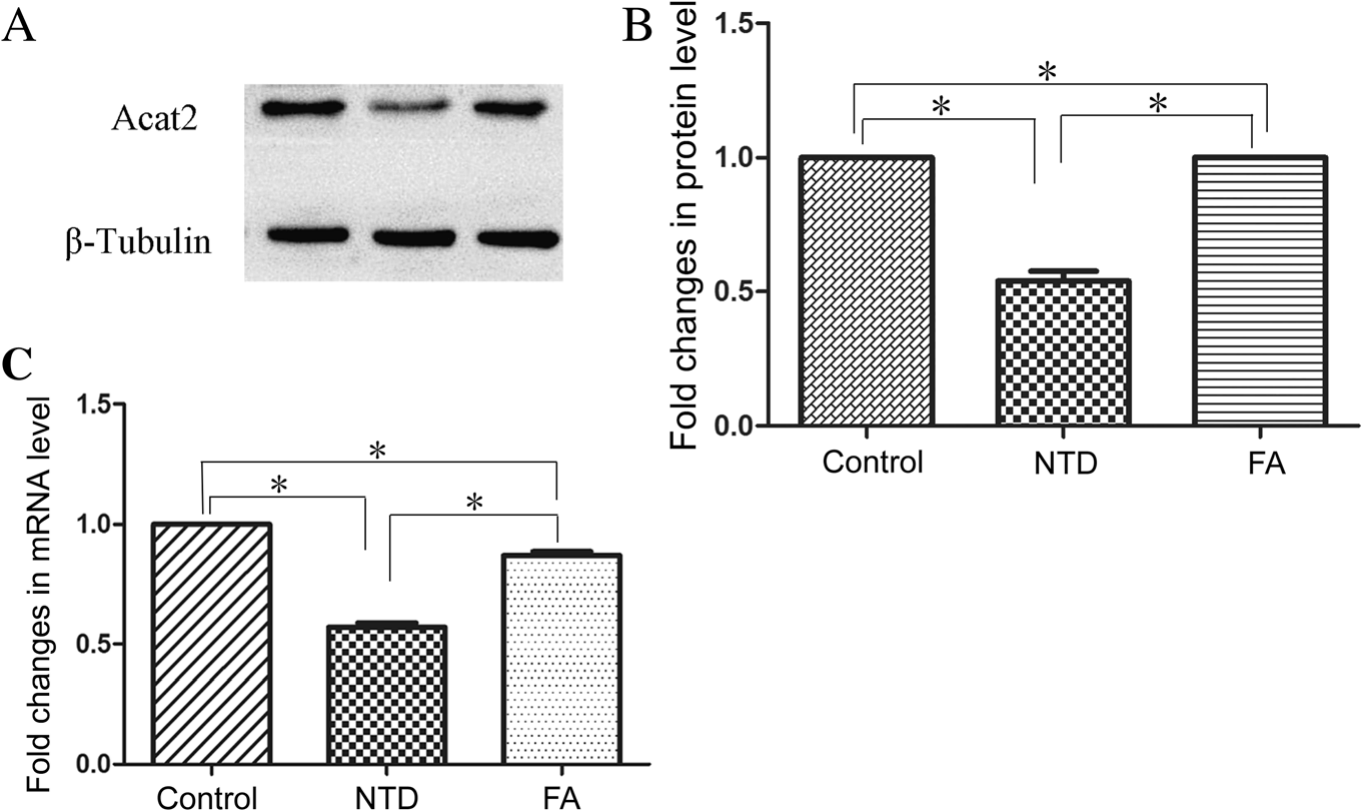
**a**, **b** Western blot analyses comparing the ACat2 protein expression in NSCs in 3 groups. **c** qRT-PCR analyses of ACat2 mRNA expression in NSCs in 3 groups. *P* < 0.05

The relative expression of ACat2 protein and mRNA was determined by Western blot (Fig. 9a and b) and qRT-PCR analyses (Fig. 9c), respectively. The ACat2 protein and mRNA level in NTD group was significantly reduced compared with healthy controls (*P* < 0.05), and was significantly increased after FA fortification (*P* < 0.05). The ACat2 protein and mRNA expression in FA group was significantly lower compared with controls (*P* < 0.05). Spearman rank correlation test showed that the H3K27me3 expression was negatively correlated with ACat2 level (rs = −0.879, *P* <0.01)

## Discussion

Epigenetic modifications such as methylation of histone H3 on lysine 27 is crucial for the regulation of gene expression. While the H3K27me3 is a signal for gene silencing, KDM6B/JMJD3, the H3K27me3 demethylase, KDM6B regulates the methylation equilibrium of H3K27me3, and thus plays an important role during the development of embryonic neural tube [20]. Recently, H3K27me3 have been gradually recognized as an epigenetic marker during neuronal differentiation. Chou et al. have found that the epigenetic regulation of chromatin structure during neurons formation is achieved through H3K27me3 and gene silencing of DNA methyltransferase [21]. In a chromatin immunoprecipitation analyses of dorsal root ganglion cell lines Hel1 ND7, H3K27 methylation was identified as an epigenetic marker for the differentiation of sensory neurons from dorsal root ganglia [22].

We have previously identified an association between ACat2, a gene related to folate metabolism and H3K27me3 by ChIP-seq technology (data not shown). Nevertheless, the mechanism between H3K27me3 and ACat2 gene in NTD has been seldom studied. In this study, we investigated the effect of FA fortification on H3K27me3 expression as well as the association between H3K27me3 and ACat2 in an ATRA-induced NTD rat model. It was shown that when compared with normal controls, FA level in neural tube was decreased (*P* <0.05), and the ability of cell proliferation and differentiation was significantly reduced (*P* <0.05). Moreover, H3K27me3 expression in NTD group was higher, and ACat2 mRNA expression was lower compared with controls (*P* <0.05). FA fortification notably reversed these changes. Additionally, H3K27me3 expression was negatively correlated with both the FA level (rs = −0.908, *P* <0.01) and ACat2 level (rs = −0.879, *P* <0.01) in the neural tube. These results suggested that FA might decrease the H3K27me3 expression in NTD through regulation on the KDM6B demethylase, and thus reduce the methylation of ACat2 gene that is involved in the folate metabolic pathway, leading to increased ACat2 expression. As a result, the stimulated ACat2 expression improved the disordered FA metabolism and thereby provided sufficient level of methyl to ensure the normal proliferation of NSCs. Consistently, Shunsuke et al. also suggested a link between the protective effect of FA on NSCs and H3K27 methylation in Sp2H mutant (pax3 - / -) mouse embryos [23]. They have suggested that FA stimulates the KDM6B level, and thus inhibits the H3K27 methylation located in the promoter region of Hes1 and Neurog2, key regulatory genes in neuronal development, leading to enhanced proliferative potential of NSCs.

## Conclusion

In summary, the increased H3K27me3 expression might cause a disorder of folate metabolic pathway by silencing ACat2 expression, leading to reduced proliferation and differentiation of NSCs, and ultimately the occurrence of NTD. FA supplementation may reverse the process and exert a protective effect on NTD.

## Abbreviations

NTD: Neural tube defect
H3K27me3: Histone H3 trimethyl Lys27
SAM: S-adenosylmethionine
FA: Folic acid
ACat2: Acyl-coenzyme a-cholesterol acyltransferase 2
HHcy: Hyperhomocysteinemia
ATRA: All-trans retinoic acid
NSC: Neural stem cells
